# Supplementary material for: An injury-induced serotonergic neuron subpopulation contributes to axon regrowth and function restoration after spinal cord injury in zebrafish
Source: Nat Commun. 2021 Dec 7;12:7093. doi: 10.1038/s41467-021-27419-w (PMC8651775; doi:10.1038/s41467-021-27419-w)
Supplement: Supplementary file 1 — Supplementary Information [file 41467_2021_27419_MOESM1_ESM.pdf]

Supplementary information:

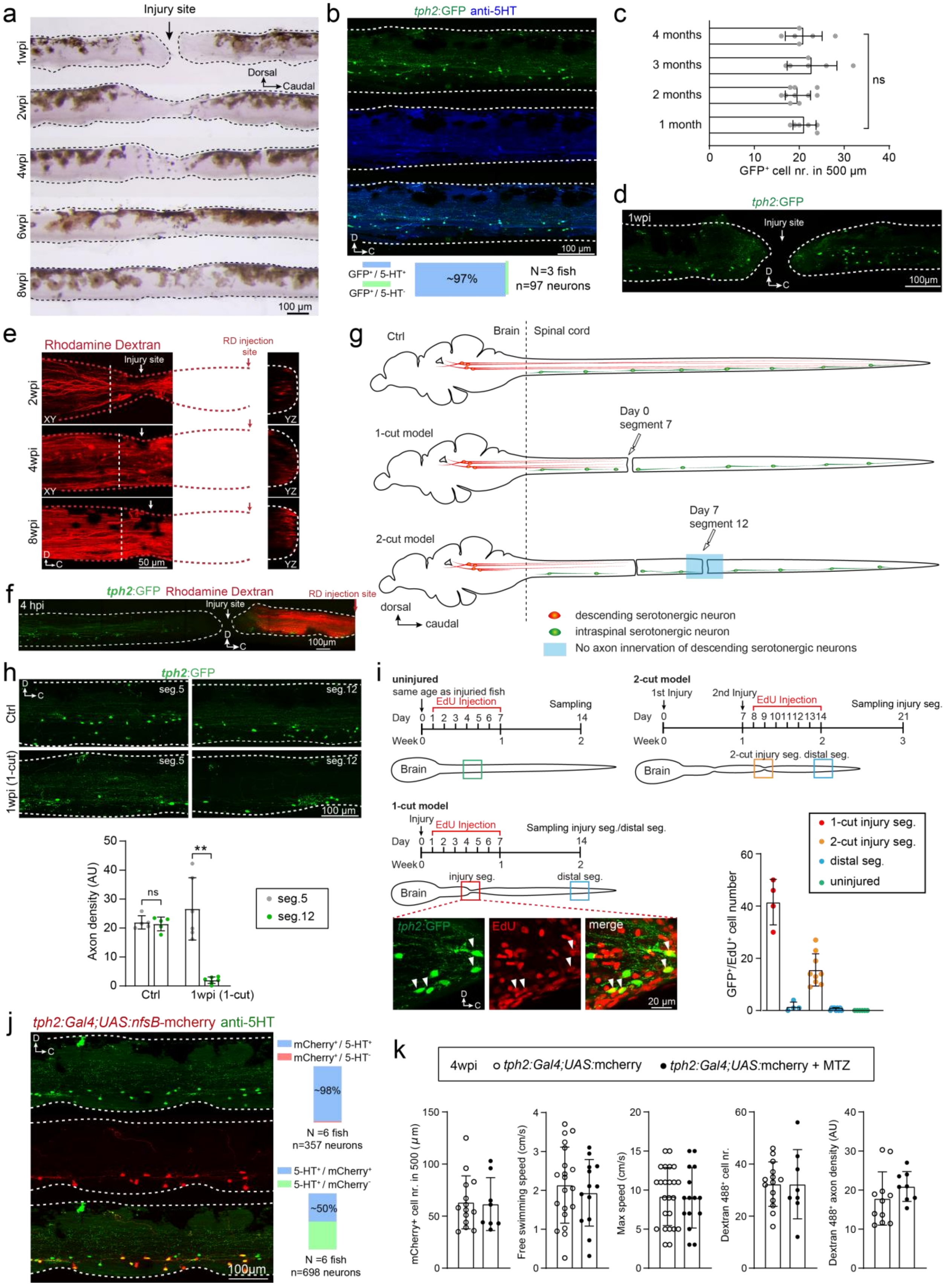
a
Injury site
1wpi
2wpi
4wpi
6wpi
8wpi
Dorsal
Caudal
100 µm
b
tph2:GFP anti-5HT
100 µm
GFP⁺/ 5-HT⁺
GFP⁺/ 5-HT⁻
~97%
N=3 fish
n=97 neurons
c
4 months
3 months
2 months
1 month
ns
GFP⁺ cell nr. in 500 µm
d
tph2:GFP
1wpi
Injury site
100µm
e
Rhodamine Dextran
RD injection site
Injury site
2wpi
4wpi
8wpi
XY
YZ
50 µm
f
tph2:GFP Rhodamine Dextran
RD injection site
4 hpi
Injury site
100µm
g
Ctrl
Brain
Spinal cord
1-cut model
Day 0
segment 7
2-cut model
Day 7
segment 12
dorsal
caudal
descending serotonergic neuron
intraspinal serotonergic neuron
No axon innervation of descending serotonergic neurons
h
tph2:GFP
Ctrl
1wpi (1-cut)
seg.5
seg.12
100 µm
Axon density (AU)
ns
**
Ctrl
1wpi (1-cut)
i
uninjured
same age as injuried fish
EdU Injection
Sampling
Day
Week
Brain
2-cut model
1st Injury
2nd Injury
Sampling injury seg.
2-cut injury seg. distal seg.
1-cut model
Injury
Sampling injury seg./distal seg.
injury seg.
distal seg.
tph2:GFP
EdU
merge
20 µm
1-cut injury seg.
2-cut injury seg.
distal seg.
uninjured
GFP⁺/EdU⁺ cell number
j
tph2:Gal4;UAS:nfsB-mcherry anti-5HT
mCherry⁺/ 5-HT⁺
mCherry⁺/ 5-HT⁻
~98%
N =6 fish
n=357 neurons
5-HT⁺/ mCherry⁺
5-HT⁺/ mCherry⁻
~50%
N =6 fish
n=698 neurons
100µm
k
4wpi
tph2:Gal4;UAS:mcherry
tph2:Gal4;UAS:mcherry + MTZ
mCherry+ cell nr. in 500 (µm)
Free swimming speed (cm/s)
Max speed (cm/s)
Dextran 488⁺ cell nr.
Dextran 488⁺ axon density (AU)

2

Supplementary Fig. 1. a Images of laterally mounted spinal cord showing the tissue recovery after SCI. **b** Immunohistochemistry images (upper) and quantification (lower) showing co-labelling of GFP$^+$ neurons with anti-serotonin antibody in the uninjured *Tg(tph2:GFP)* fish. **c** Quantification of GFP$^+$ ISNs numbers in a 500 μm region of spinal cord in 1-, 2-, 3- and 4-months old uninjured fish. **d** Immunohistochemistry images show GFP$^+$ ISNs distribution at 1 week post injury (wpi) in SCI *Tg(tph2:GFP)* fish. **e** Immunohistochemistry images show the Rhodamine Dextran (RD) retrograde labeling of axon-regrown spinal interneurons after SCI. The regrown axon density was calculated via Fiji's YZ orthogonal view. The RD was applied at two segments caudal to the injury site as indicated by red arrows. **f** Images show the RD retrograde labeling at 1 wpi in SCI *Tg(tph2:GFP)* fish. No spinal interneuron rostral to the injury site was labeled when the spinal cord tissue was still separated. **g** Illustration of injury models showing the distribution of braining descending and intraspinal serotonergic neurons and 1-cut/2-cut transection locations. The spinal condition with no descending serotonergic axon innervation is indicated by the blue box. **h** (upper) Immunohistochemistry images show the descending GFP$^+$ serotonergic axon density at 5th segment (two segments rostral to the 1-cut injury site) and 12th segment (five segments caudal to the 1-cut injury site) at 1wpi in SCI fish and at the corresponding segments in uninjured animals. (lower) Mean data of GFP$^+$ axon density of 5th segment and 12th segment regions in uninjured and 1 wpi fish. **i** (upper) Protocols of EdU intraperitoneal injection and observation sites to monitor regeneration of ISNs in the uninjured, 1-cut and 2-cut SCI *Tg(tph2:GFP)* line. (bottom left) The immunohistochemistry images represent a single plane of the area bounded by the red box showing EdU co-labeled with *tph2*:GFP (arrowheads), indicating regeneration of ISNs at the injury segment. (bottom right) Mean data of GFP$^+$/EdU$^+$ cell numbers in the injury segment or distal segments of 1-cut and 2-cut animals and corresponding segments in uninjured fish. **j** Immunohistochemistry images (left) and quantification (right) of co-labeled mCherry$^+$ neurons with anti-serotonin in the uninjured *Tg(tph2:Gal4;UAS:nfsB-mCherry)* line. **k** Quantification of mCherry$^+$ ISNs numbers in the region of 500 μm covering the injury site; free-swimming speed; maximum swimming speed; retrogradely labeled axon-regrown interneuron numbers and regrown axon density after SCI with/without MTZ treatment in *Tg(tph2:Gal4;UAS:mCherry)* fish. Images of a, d, e, f represent results from over ten independent experiments. All data are presented as mean ± SD. *, P <0.05, **, P <0.01, ***, P <0.001, ****, P <0.0001, significant difference. For detailed statistics, see Supplementary Table 1.

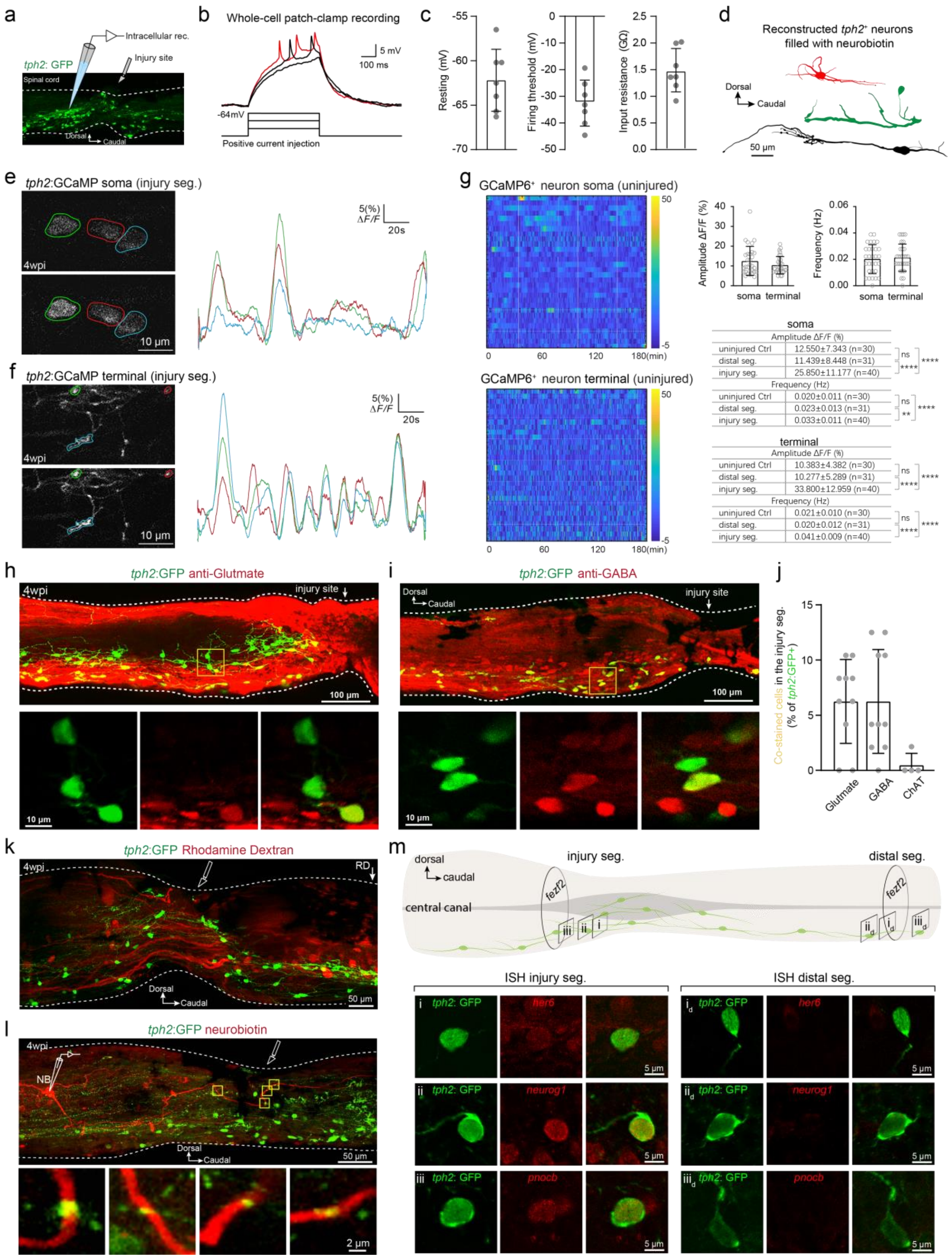

Supplementary Fig. 2. **a** Illustration of whole-cell patch-clamp recording of GFP$^+$ ISNs in the injury segment performed in *Tg(tph2:GFP)* fish. **b** Action potentials evoked by injection of depolarizing current pulses in GFP$^+$ ISNs in the injury segment at 2 wpi. **c** Mean data of resting

potential, firing threshold and input resistance of GFP$^+$ ISNs in the injury segment. **d** Reconstruction of morphology of three typical ISNs labelled by intracellular injection of neurobiotin. **e-f** (left) Images showing typical maximal intracellular Ca^{2+} increases (bottom) from the resting level (top) and (right) Ca^{2+} changes over time in the outlined GCaMP6$^+$ ISNs soma (**e**) and axon varicosities (**f**) of the injury segment of *Tg(tph2:Gal4;UAS:GCaMP6)* line at 4 wpi. **g** (left) Heatmaps showing Ca^{2+} oscillation during 3 min of GCaMP6$^+$ ISNs soma and terminals in the uninjured fish. (right upper) Quantification of Ca^{2+} oscillation amplitude and frequency of GCaMP6$^+$ ISNs soma and terminal in the uninjured fish. (right bottom) Comparison of Ca^{2+} oscillation amplitude and frequency among the injury segment, distal segments and uninjured control. **h-i** Immunohistochemistry images show the staining of anti-Glutamate (**h**) or anti-GABA (**i**) using *Tg(tph2:GFP)* line at 4 wpi. Expanded images represent a single plane of the area bounded by the yellow boxes showing co-labeled cells. **j** Graph shows the percentage of *tph2:GFP*$^+$ neurons co-staining for glutamate, GABA and ChAT in the injury segment. **k** Immunohistochemistry images of *Tg(tph2:GFP)* fish spinal cord retrogradely labeled with RD at 4 wpi showing that regenerated ISNs are in close apposition to re-growing axons. **l** (top) Immunohistochemistry images show a single retrogradely labeled axon-regrown spinal interneuron reconstructed by intracellular injection of neurobiotin in the injury segment of *Tg(tph2:GFP)* line at 4 wpi. (bottom) Enlarged yellow boxes highlight the co-localization of re-growing axon of spinal interneurons and GFP$^+$ ISNs terminals. **m** Illustration of cross-sectioning positions (upper) and images (lower) show *in situ* hybridization (ISH) for *her6*, *neurog1* and *pnocb* in GFP$^+$ ISNs in the injury and distal spinal segments in *Tg*(*tph2*:GFP) line, representing results from three independent experiments. Sections were co-stained using anti-GFP antibody. Images of e, f, h, i, k, l represent results from over ten independent experiments. All data are presented as mean ± SD. *, P <0.05, **, P <0.01, ***, P <0.001, ****, P <0.0001, significant difference. For detailed statistics, see Supplementary Table 1.

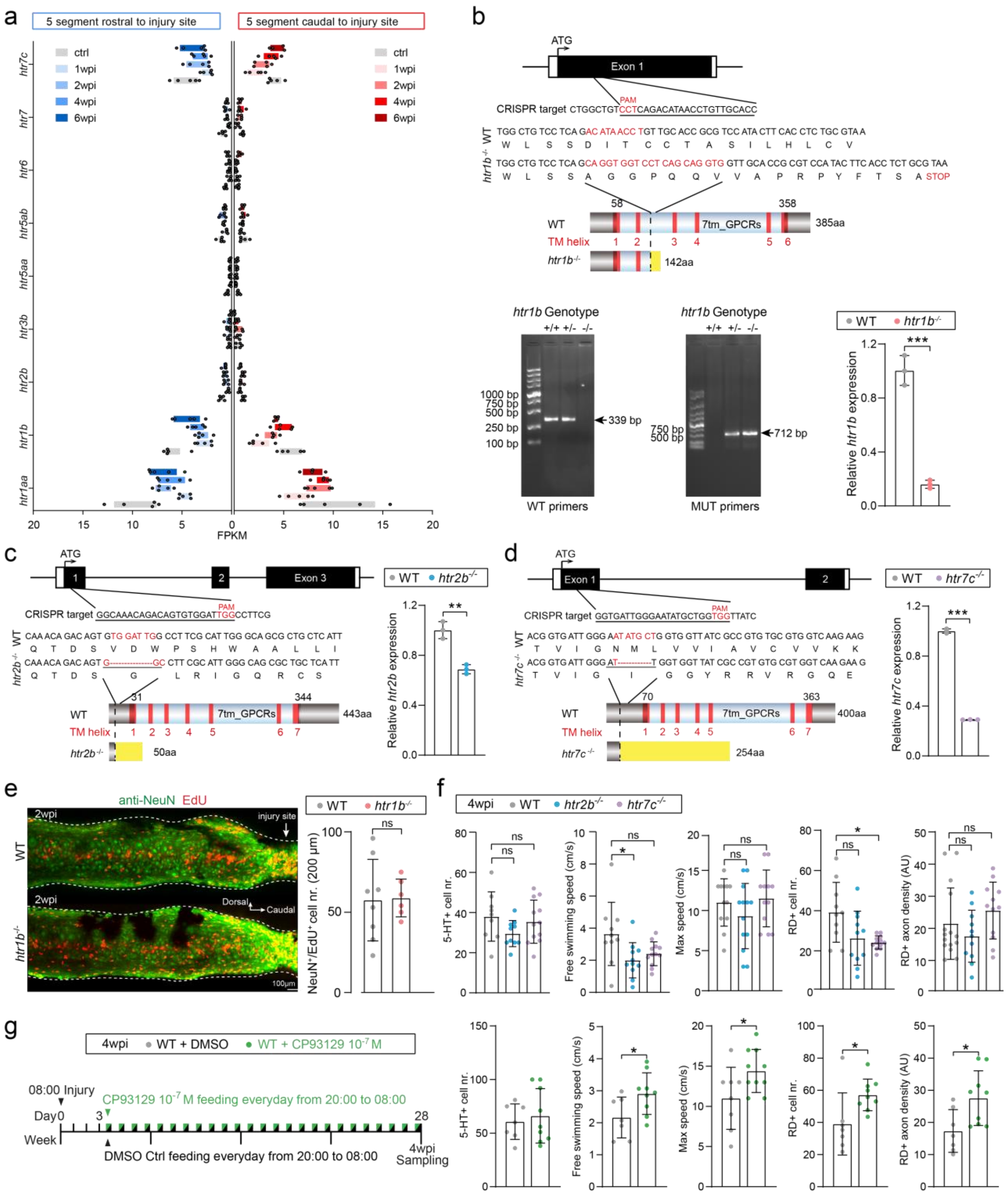

Supplementary Fig. 3. a Mean FPKM values of differentially expressed 5-HT receptor genes in the rostral and caudal five spinal segments after SCI compared to those in uninjured fish (n=3 fish in each group, N = 4-5 libraries of pooled samples). **b** Generation of *htr1b* mutant. Top panel shows the Htr1b protein containing a 7tm_GPCRs domain (385 aa), and the truncated short peptide of *htr1b* mutant (142 aa). The CRISPR target site containing a PAM sequence is indicated; mutated

nucleotides and amino acids are shown in red. Lower left panel shows typical genotyping blots of wild-type, *htr1b* heterozygous (*htr1b*$^{+/-}$), and homozygous mutant (*htr1b*$^{-/-}$) animals. Specific primer sets containing WT or MUT sequences were used for specific detection of the wild-type (335 bp) and mutant (712 bp) alleles. Lower right panel shows decreased *htr1b* expression in *htr1b*$^{-/-}$ animals compared to wild-type controls. Relative expression was normalized to *gapdh* levels. **c** Generation of *htr2b* mutant. (left) The Htr2b protein containing a 7tm_GPCRs domain (443 aa), and the truncated short peptide of *htr1b* mutant (50 aa). CRISPR target site containing a PAM sequence is indicated; mutated nucleotides and amino acids are shown in red. (right) QRT-PCR shows decreased *htr2b* expression in *htr2b*$^{-/-}$ animals compared to wild-type controls. Relative expression was normalized to *gapdh* levels. **d** Generation of *htr7c* mutant. (left) The Htr7c protein containing a 7tm_GPCRs domain (400 aa), and the truncated short peptide of *htr7c* mutant (254 aa). CRISPR target site containing a PAM sequence is indicated; mutated nucleotides and amino acids are shown in red. (right) QRT-PCR shows decreased *htr7c* expression in *htr7c*$^{-/-}$ animals compared to wild-type controls. Relative expression was normalized to *gapdh* levels. **e** (Left) Immunohistochemistry images show regenerated neurons (co-labeled by EdU and anti-NeuN) in the spinal cord of WT and *htr1b*$^{-/-}$ animals at 2wpi. (Right) Mean numbers of regenerated neurons in WT and *htr1b*$^{-/-}$ animals at 2wpi were not significantly different. **f** Mean data for WT (grey), *htr2b*$^{-/-}$ (blue) and *htr7c*$^{-/-}$ (purple) SCI fish ISNs numbers in a 500 μm region covering injury site; free swimming speed; maximum speed; axon-regrown spinal interneuron numbers and regrown axon density at 4 wpi. **g** (left) Protocol for administration of the 5-HT_{1B} agonist CP93129 dihydrochloride and sampling after SCI in wild-type animals. (right) Quantification of ISNs numbers in a 500 μm region covering the injury site; free swimming speed; maximum speed; axon-regrown spinal interneuron numbers; regrown axon density in SCI fish treated with DMSO (grey dots) and CP93129 (green dots). All data are presented as mean ± SD. *, $P < 0.05$, **, $P < 0.01$, ***, $P < 0.001$, ****, $P < 0.0001$, significant difference. For detailed statistics, see Supplementary Table 1.

Supplementary Table 1. Detailed statistics

Figure	Statistics	Result	Post-hoc Test	Comparison	Significance	*P* value
**Fig 1b**	One-way ANOVA	F(5, 37.86)=26.45, P < 0.0001	Dunnett's T3 test	Ctrl. vs. 1wpi	****	*P*adj < 0.0001
				Ctrl. vs. 2wpi	ns	*P*adj = 0.6508
				Ctrl. vs. 4wpi	****	*P*adj < 0.0001
				Ctrl. vs. 6wpi	***	*P*adj = 0.0005
				Ctrl. vs.8wpi	*	*P*adj = 0.0107
				1wpi vs. 2wpi	***	*P*adj = 0.0007
				1wpi vs. 4wpi	****	*P*adj < 0.0001
				1wpi vs. 6wpi	****	*P*adj < 0.0001
				1wpi vs. 8wpi	**	*P*adj = 0.0011
				2wpi vs. 4wpi	****	*P*adj < 0.0001
				2wpi vs. 6wpi	**	*P*adj = 0.0024
				2wpi vs. 8wpi	*	*P*adj = 0.0466
				4wpi vs. 6wpi	ns	*P*adj > 0.9999
				4wpi vs. 8wpi	ns	*P*adj = 0.9993
				6wpi vs. 8wpi	ns	*P*adj = 0.9796
	Descriptive	Ctrl: 19.70 ± 2.791 (n = 10 fish)				
		1wpi: 8.692 ± 3.425 (n = 13 fish)				
		2wpi: 25.44 ± 12.02 (n = 16 fish)				
		4wpi: 53.36 ± 14.05 (n = 14 fish)				
		6wpi: 57.46 ± 21.87 (n = 13 fish)				
		8wpi: 48.00 ± 18.14 (n = 10 fish)				
**Fig 1c**	One-way ANOVA	F(5, 50.40)=46.34, P < 0.0001	Dunnett's T3 test	Ctrl. vs. 1wpi	****	*P*adj < 0.0001
				Ctrl. vs. 2wpi	****	*P*adj < 0.0001
				Ctrl. vs. 4wpi	****	*P*adj < 0.0001
				Ctrl. vs. 6wpi	****	*P*adj < 0.0001
				Ctrl. vs.8wpi	ns	*P*adj = 0.2871
				1wpi vs. 2wpi	***	*P*adj = 0.0003
				1wpi vs. 4wpi	****	*P*adj < 0.0001
				1wpi vs. 6wpi	****	*P*adj < 0.0001
				1wpi vs. 8wpi	****	*P*adj < 0.0001
				2wpi vs. 4wpi	**	*P*adj = 0.0084
				2wpi vs. 6wpi	***	*P*adj = 0.0006
				2wpi vs. 8wpi	****	*P*adj < 0.0001
				4wpi vs. 6wpi	ns	*P*adj > 0.9999
				4wpi vs. 8wpi	ns	*P*adj = 0.0647
				6wpi vs. 8wpi	ns	*P*adj = 0.1425
	Descriptive	Ctrl: 2.313 ± 0.240 (n = 15 fish)				
		1wpi: 0.3289 ± 0.06248 (n = 10 fish)				
		2wpi: 0.7058 ± 0.1982 (n = 12 fish)				
		4wpi: 1.339 ± 0.5224 (n = 14 fish)				
		6wpi: 1.436 ± 0.4857 (n = 15 fish)				
		8wpi: 1.946 ± 0.5391 (n = 15 fish)				
**Fig 1d**	One-way ANOVA	F(4, 32.32)=14.85, P < 0.0001	Dunnett's T3 test	Ctrl vs. 2wpi	****	*P*adj < 0.0001
				Ctrl vs. 4wpi	****	*P*adj < 0.0001
				Ctrl vs. 6wpi	*	*P*adj = 0.0343

				Ctrl vs. 8wpi	ns	*P*adj = 0.2442
				2wpi vs. 4wpi	***	*P*adj = 0.0005
				2wpi vs. 6wpi	**	*P*adj = 0.0043
				2wpi vs. 8wpi	**	*P*adj = 0.0075
				4wpi vs. 6wpi	ns	*P*adj = 0.1856
				4wpi vs. 8wpi	ns	*P*adj = 0.1793
				6wpi vs. 8wpi	ns	*P*adj > 0.9999
	Descriptive	Ctrl: 21.00 ± 2.687 (n = 10 fish)				
		2wpi: 5.654 ± 1.586 (n = 13 fish)				
		4wpi: 9.385 ± 2.181 (n = 13 fish)				
		6wpi: 14.25 ± 6.886 (n = 14 fish)				
		8wpi: 15.33 ± 8.797 (n = 15 fish)				
**Fig 1h RD+ cell**	One-way ANOVA	F(2, 23.76)=5.708, P = 0.0095	Dunnett's T3 test	2wpi vs. 4wpi	***	*P*adj = 0.0004
				2wpi vs. 8wpi	*	*P*adj = 0.0143
				4wpi vs. 8wpi	ns	*P*adj = 0.9961
	Descriptive	2wpi: 22.33 ± 5.007 (n = 6 fish)				
		4wpi: 39.08 ± 9.070 (n = 12 fish)				
		8wpi: 38.07 ± 16.53 (n = 14 fish)				
**Fig 1h RD+ axon**	One-way ANOVA	F(2, 26.46)=13.76, P < 0.0001	Dunnett's T3 test	2wpi vs. 4wpi	*	*P*adj = 0.0412
				2wpi vs. 8wpi	****	*P*adj < 0.0001
				4wpi vs. 8wpi	ns	*P*adj = 0.1177
	Descriptive	2wpi: 14.05 ± 4.344 (n = 13 fish)				
		4wpi: 23.30 ± 11.86 (n = 14 fish)				
		8wpi: 31.56 ± 7.794 (n = 13 fish)				
**Fig 1l GFP+ cell**	One-way ANOVA	F(2, 13.05)=20.72, P < 0.0001	Dunnett's T3 test	Ctrl vs. 2wpi	****	*P*adj < 0.0001
				Ctrl vs. 4wpi	***	*P*adj = 0.0008
				2wpi vs. 4wpi	*	*P*adj = 0.0235
	Descriptive	Ctrl: 30.13 ± 6.978 (n = 15 fish)				
		2wpi: 42.88 ± 7.302 (n = 16 fish)				
		4wpi: 62.00 ± 16.60 (n = 9 fish)				
**Fig 1l RD+ cell**	Unpaired *t*-test (Two-tailed)	t=2.288, df=27		2wpi vs. 4wpi	*	*P* = 0.0302
	Descriptive	2wpi: 31.69 ± 13.28 (n = 13 fish)				
		4wpi: 47.44 ± 21.69 (n = 16 fish)				
**Fig 1l RD+ axon**	Unpaired *t*-test (Two-tailed)	t=3.333, df=23		2wpi vs. 4wpi	**	*P* = 0.0029
	Descriptive	2wpi: 10.51 ± 6.796 (n = 12 fish)				
		4wpi: 23.54 ± 11.85 (n = 13 fish)				
**Fig 2c**	Unpaired *t*-test with Welch's correction (Two-tailed)	t=5.218, df=11.58		2wpi: MTZ- vs. MTZ+	***	*P* = 0.0002
	Unpaired *t*-test with Welch's correction (Two-tailed)	t=7.666, df=4.463		4wpi: MTZ- vs. MTZ+	***	P = 0.001
	Descriptive	2wpi MTZ-: 22.00 ± 12.91 (n = 12 fish)				
		2wpi MTZ+: 2.294 ± 2.494 (n = 17 fish)				
		4wpi MTZ-: 24.80 ± 6.058 (n = 5 fish)				
		4wpi MTZ+: 3.444 ± 1.944 (n = 9 fish)				

**Fig 2d**	Unpaired *t*-test with Welch's correction (Two-tailed)	t=5.015, df=9.942		2wpi: MTZ- vs. MTZ+	***	*P* = 0.0005
	Unpaired *t*-test with Welch's correction (Two-tailed)	t=6.697, df=17.64		4wpi: MTZ- vs. MTZ+	****	*P* < 0.0001
	Descriptive	2wpi MTZ-: 2.208 ± 0.984 (n = 10 fish)				
		2wpi MTZ+: 0.6071 ± 0.3390 (n = 23 fish)				
		4wpi MTZ-: 3.408 ± 1.526 (n = 16 fish)				
		4wpi MTZ+: 0.7415 ± 0.4678 (n = 17 fish)				
**Fig 2e**	Unpaired *t*-test (Two-tailed)	t=2.734, df=24		2wpi: MTZ- vs. MTZ+	*	*P* = 0.0116
	Unpaired *t*-test (Two-tailed)	t=2.434, df=17		4wpi: MTZ- vs. MTZ+	*	*P* = 0.0262
	Descriptive	2wpi MTZ-: 11.10 ± 2.998 (n = 10 fish)				
		2wpi MTZ+: 7.625 ± 3.243 (n = 16 fish)				
		4wpi MTZ-: 12.50 ± 3.209 (n = 6 fish)				
		4wpi MTZ+: 9.462 ± 2.184 (n = 13 fish)				
**Fig 2f**	Unpaired *t*-test (Two-tailed)	t=3.049	df=27	2wpi: MTZ- vs. MTZ+	**	*P* = 0.0051
	Unpaired *t*-test (Two-tailed)	t=2.241	df=12	4wpi: MTZ- vs. MTZ+	*	*P* = 0.0447
	Descriptive	2wpi MTZ-: 23.25 ± 11.76 (n = 12 fish)				
		2wpi MTZ+: 11.76 ± 8.562 (n = 17 fish)				
		4wpi MTZ-: 33.00 ± 3.937 (n = 5 fish)				
		4wpi MTZ+: 22.89 ± 9.506 (n = 9 fish)				
**Fig 2g**	Unpaired *t*-test with Welch's correction (Two-tailed)	t=3.298, df=13.91		2wpi: MTZ- vs. MTZ+	**	*P* = 0.0053
	Unpaired *t*-test (Two-tailed)	t=4.606, df=12		4wpi: MTZ- vs. MTZ+	***	*P* = 0.0006
	Descriptive	2wpi MTZ-: 25.34 ± 15.48 (n = 12 fish)				
		2wpi MTZ+: 9.662 ± 6.672 (n = 17 fish)				
		4wpi MTZ-: 40.35 ± 7.236 (n = 5 fish)				
		4wpi MTZ+: 19.00 ± 8.799 (n = 9 fish)				
**Fig 2i**	Unpaired *t*-test (Two-tailed)	t=6.096, df=21		2wpi: MTZ- vs. MTZ+	****	*P* < 0.0001
	Unpaired *t*-test with Welch's correction (Two-tailed)	t=5.513, df=12.54		4wpi: MTZ- vs. MTZ+	***	*P* = 0.0001
	Descriptive	2wpi MTZ-: 7.636 ± 2.203 (n = 11 fish)				
		2wpi MTZ+: 2.333 ± 1.969 (n = 12 fish)				
		4wpi MTZ-: 11.83 ± 5.967 (n = 12 fish)				
		4wpi MTZ+: 2.000 ± 1.309 (n = 8 fish)				
**Fig 2j**	Unpaired *t*-test with Welch's correction (Two-tailed)	t=1.332, df=7.233		2wpi: MTZ- vs. MTZ+	ns	*P* = 0.2233
	Unpaired *t*-test (Two-tailed)	t=2.422, df=16		4wpi: MTZ- vs. MTZ+	*	*P* = 0.0277
	Descriptive	2wpi MTZ-: 1.271 ± 1.186 (n = 7 fish)				
		2wpi MTZ+: 0.6443 ± 0.4068 (n = 8 fish)				

		4wpi MTZ-: 1.424 ± 0.5493 (n = 10 fish)			
		4wpi MTZ+: 0.7940 ± 0.5471 (n = 8 fish)			
**Fig 2k**	Unpaired *t*-test (Two-tailed)	t=2.178, df=25	2wpi: MTZ- vs. MTZ+	*	*P* = 0.0391
	Unpaired *t*-test with Welch's correction (Two-tailed)	t=3.464, df=20.74	4wpi: MTZ- vs. MTZ+	**	*P* = 0.0024
	Descriptive	2wpi MTZ-: 7.083 ± 3.423 (n = 12 fish)			
		2wpi MTZ+: 4.867 ± 1.767 (n = 15 fish)			
		4wpi MTZ-: 8.125 ± 3.575 (n = 16 fish)			
		4wpi MTZ+: 4.700 ± 1.337 (n = 10 fish)			
**Fig 2l**	Unpaired *t*-test (Two-tailed)	t=2.983, df=21	2wpi: MTZ- vs. MTZ+	**	*P* = 0.0071
	Unpaired *t*-test (Two-tailed)	t=2.146, df=18	4wpi: MTZ- vs. MTZ+	*	*P* = 0.0458
	Descriptive	2wpi MTZ-: 11.91 ± 4.929 (n = 11 fish)			
		2wpi MTZ+: 6.083 ± 4.441 (n = 12 fish)			
		4wpi MTZ-: 17.17 ± 6.013 (n = 12 fish)			
		4wpi MTZ+: 11.38 ± 5.755 (n = 8 fish)			
**Fig 2m**	Unpaired *t*-test (Two-tailed)	t=2.368, df=22	2wpi: MTZ- vs. MTZ+	*	*P* = 0.0271
	Unpaired *t*-test (Two-tailed)	t=0.7073, df=19	4wpi: MTZ- vs. MTZ+	ns	*P* = 0.4880
	Descriptive	2wpi MTZ-: 7.307 ± 2.332 (n = 12 fish)			
		2wpi MTZ+: 4.807 ± 2.817 (n = 12 fish)			
		4wpi MTZ-: 22.09 ± 12.45 (n = 12 fish)			
		4wpi MTZ+: 18.63 ± 8.953 (n = 9 fish)			
**Fig 3d**	Unpaired *t*-test with Welch's correction (Two-tailed)	t=17.95, df=23.60	injury seg. vs. distal seg.	****	*P* < 0.0001
	Descriptive	injury seg.: 5.738 ± 0.9833 (n = 20 neurons examined over 4 independent experiments)			
		distal seg.: 1.557 ± 0.3445 (n = 20 neurons examined over 4 independent experiments)			
**Fig 3h amplitude**	Unpaired *t*-test (Two-tailed)	t=5.974, df=69	soma: injury seg. vs. distal seg.	****	*P* < 0.0001
	Unpaired *t*-test with Welch's correction (Two-tailed)	t=10.42, df=54.30	terminal: injury seg. vs. distal seg.	****	*P* < 0.0001
	Descriptive	soma injury seg.: 25.85 ± 11.18 (n = 40 neurons examined over 8 independent experiments)			
		soma distal seg.: 11.44 ± 8.448 (n = 31 neurons examined over 8 independent experiments)			
		terminal injury seg.: 33.80 ± 12.96 (n = 40 neurons examined over 8 independent experiments)			
		terminal distal seg.: 10.28 ± 5.289 (n = 31 neurons examined over 8 independent experiments)			
**Fig 3h frequency**	Unpaired *t*-test (Two-tailed)	t=3.596, df=69	soma: injury seg. vs. distal seg.	***	*P* = 0.0006
	Unpaired *t*-test (Two-tailed)	t=8.250, df=69	terminal: injury seg. vs. distal seg.	****	*P* < 0.0001
	Descriptive	soma injury seg.: 0.03292 ± 0.01085 (n = 40 neurons examined over 8 independent experiments)			
		soma distal seg.: 0.02258 ± 0.01337 (n = 31 neurons examined over 8 independent experiments)			
		terminal injury seg.: 0.04069 ± 0.009106 (n = 40 neurons examined over 8 independent experiments)			
		terminal distal seg.: 0.02043 ± 0.01160 (n = 31 neurons examined over 8 independent experiments)			
**Fig 3i**	Paired *t*-test (Two-tailed)	t=10.29, df=2	injury seg. vs. distal seg.	**	*P* = 0.0093

	Descriptive	injury seg.: 0.09034 ± 0.01658 (n = 3 independent experiments)			
		distal seg.: 0.02877 ± 0.01152 (n = 3 independent experiments)			
**Fig 3q**	Paired *t*-test (Two-tailed)	t=6.318, df=4.463	Mibefradil- vs. Mibefradil+	**	*P* = 0.0032
	Descriptive	Mibefradil-: 171.6 ± 61.12 (n = 3 independent experiments)			
		Mibefradil+: 6.180 ± 2.657 (n = 3 independent experiments)			
**Fig 4d 5-HT+ cell**	Unpaired *t*-test (Two-tailed)	t=1.025, df=16	2wpi: WT vs. *htr1b-/-*	ns	*P* = 0.3204
	Unpaired *t*-test (Two-tailed)	t=0.1349, df=18	4wpi: WT vs. *htr1b-/-*	ns	*P* = 0.8942
	Descriptive	2wpi WT: 42.13 ± 12.55 (n = 8 fish)			
		2wpi *htr1b-/-*: 36.20 ± 11.89 (n = 10 fish)			
		4wpi WT: 38.00 ± 12.27 (n = 9 fish)			
		4wpi *htr1b-/-*: 37.27 ± 11.77 (n = 11 fish)			
**Fig 4d Free swimming**	Unpaired *t*-test (Two-tailed)	t=2.673, df=14	2wpi: WT vs. *htr1b-/-*	*	*P* = 0.0182
	Unpaired *t*-test with Welch's correction (Two-tailed)	t=3.272, df=10.77	4wpi: WT vs. *htr1b-/-*	**	*P* = 0.0076
	Descriptive	2wpi WT: 2.306 ± 1.099 (n = 7 fish)			
		2wpi *htr1b-/-*: 1.193 ± 0.5365 (n = 9 fish)			
		4wpi WT: 3.638 ± 1.968 (n = 10 fish)			
		4wpi *htr1b-/-*: 1.504 ± 0.6756 (n = 12 fish)			
**Fig 4d Max speed**	Unpaired *t*-test (Two-tailed)	t=3.143, df=18	2wpi: WT vs. *htr1b-/-*	**	*P* = 0.0056
	Unpaired *t*-test (Two-tailed)	t=2.646, df=28	4wpi: WT vs. *htr1b-/-*	*	*P* = 0.0132
	Descriptive	2wpi WT: 8.000 ± 1.944 (n = 10 fish)			
		2wpi *htr1b-/-*: 5.000 ± 2.309 (n = 10 fish)			
		4wpi WT: 11.00 ± 2.954 (n = 12 fish)			
		4wpi *htr1b-/-*: 7.667 ± 3.630 (n = 18 fish)			
**Fig 4d RD+ cell**	Unpaired *t*-test (Two-tailed)	t=2.149, df=17	2wpi: WT vs. *htr1b-/-*	*	*P* = 0.0464
	Unpaired *t*-test (Two-tailed)	t=4.158, df=19	4wpi: WT vs. *htr1b-/-*	***	*P* = 0.0005
	Descriptive	2wpi WT: 20.44 ± 8.338 (n = 9 fish)			
		2wpi *htr1b-/-*: 13.30 ± 6.093 (n = 10 fish)			
		4wpi WT: 39.20 ± 14.91 (n = 10 fish)			
		4wpi *htr1b-/-*: 16.55 ± 9.761 (n = 11 fish)			
**Fig 4d RD+ axon**	Unpaired *t*-test with Welch's correction (Two-tailed)	t=4.153, df=9.510	2wpi: WT vs. *htr1b-/-*	**	*P* = 0.0022
	Unpaired *t*-test with Welch's correction (Two-tailed)	t=3.847, df=18.99	4wpi: WT vs. *htr1b-/-*	**	*P* = 0.0011
	Descriptive	2wpi WT: 12.11 ± 4.476 (n = 8 fish)			
		2wpi *htr1b-/-*: 4.968 ± 2.124 (n = 10 fish)			
		4wpi WT: 21.47 ± 11.11 (n = 15 fish)			
		4wpi *htr1b-/-*: 9.403 ± 4.207 (n = 11 fish)			

**Fig 4f vglut2a**	One-way ANOVA	F(2, 26)=8.894, P = 0.0011	Tukey's test	Ctrl vs. 4wpi	***	*Padj* = 0.0008
				Ctrl vs. 8wpi	**	*Padj* = 0.0049
				4wpi vs. 8wpi	ns	*Padj* = 0.4755
	Descriptive	Ctrl: 62.03 ± 6.266 (n = 4 fish)				
		4wpi: 85.66 ± 8.346 (n = 11 fish)				
		8wpi: 81.07 ± 11.13 (n = 14 fish)				
**Fig 4f glyt2**	One-way ANOVA	F(2, 26)=36.12, P < 0.0001	Tukey's test	Ctrl vs. 4wpi	****	*Padj* < 0.0001
				Ctrl vs. 8wpi	****	Padj < 0.0001
				4wpi vs. 8wpi	ns	*Padj* = 0.9665
	Descriptive	Ctrl: 30.10 ± 6.272 (n = 4 fish)				
		4wpi: 8.522 ± 5.156 (n = 11 fish)				
		8wpi: 8.044 ± 4.011 (n = 14 fish)				
**Fig 4h**	Unpaired *t*-test (Two-tailed)	t=5.713, df=4		GFP+/RD+: Ctrl vs. 4wpi	**	*P* = 0.0046
	Unpaired *t*-test (Two-tailed)	t=3.400, df=4		GFP+/RD+: 4wpi vs. 8wpi	*	*P* = 0.0273
	Unpaired *t*-test (Two-tailed)	t=5.514, df=4		GFP+/RD+: Ctrl vs. 8wpi	**	*P* = 0.0053
	Unpaired *t*-test (Two-tailed)	t=0.1044, df=4		Ctrl: GFP+/RD- vs. GFP+/RD+	ns	*P* = 0.9219
	Unpaired *t*-test (Two-tailed)	t=5.720, df=4		4wpi: GFP+/RD- vs. GFP+/RD+	**	*P* = 0.0046
	Unpaired *t*-test (Two-tailed)	t=4.479, df=4		8wpi: GFP+/RD- vs. GFP+/RD+	*	*P* = 0.0110
	Descriptive	GFP+/RD+ Ctrl: 1.114 ± 0.4259 (n = 3 technical replicates of 40 pooled fish, the experiment was performed in triplicate)				
		GFP+/RD+ 4wpi: 3.616 ± 0.6280 (n = 3 technical replicates of 40 pooled fish, the experiment was performed in triplicate)				
		GFP+/RD+ 8wpi: 7.894 ± 2.087 (n = 3 technical replicates of 40 pooled fish, the experiment was performed in triplicate)				
		GFP+/RD- Ctrl: 1.075 ± 0.4904 (n = 3 technical replicates of 40 pooled fish, the experiment was performed in triplicate)				
		GFP+/RD- 4wpi: 1.337 ± 0.2864 (n = 3 technical replicates of 40 pooled fish, the experiment was performed in triplicate)				
		GFP+/RD- 8wpi: 2.365 ± 0.4652 (n = 3 technical replicates of 40 pooled fish, the experiment was performed in triplicate)				
**Fig 4j Firing threshold**	Unpaired *t*-test (Two-tailed)	t=2.470, df=32		WT vs. htr1b-/-	*	*P* = 0.0190
	Descriptive	WT: -16.84 ± 6.663 (n = 21 neurons examined over 21 independent experiments)				
		htr1b-/-: -24.04 ± 10.39 (n = 13 neurons examined over 13 independent experiments)				
**Fig 4j input resistance**	Unpaired *t*-test (Two-tailed)	t=2.719, df=13.91		WT vs. htr1b-/-	*	*P* = 0.0176
	Descriptive	WT: 0.07022 ± 0.04573 (n = 11 neurons examined over 11 independent experiments)				
		htr1b-/-: 0.1492 ± 0.06133 (n = 4 neurons examined over 4 independent experiments)				
**Fig 4j Resting**	Unpaired *t*-test (Two-tailed)	t=1.635, df=29		WT vs. htr1b-/-	ns	*P* = 0.1130
	Descriptive	WT: -61.90 ± 2.369 (n = 21 neurons examined over 21 independent experiments)				
		htr1b-/-: -60.57 ± 1.380 (n = 10 neurons examined over 10 independent experiments)				

**Fig 4l thickness**	Unpaired *t*-test (Two-tailed)	t=3.587, df=30		WT vs. htr1b-/-	**	*P* = 0.0012
	Descriptive	WT: 0.02065 ± 0.007073 (n = 20 neurons examined over 20 independent experiments)				
		htr1b-/-: 0.01246 ± 0.004511 (n = 12 neurons examined over 12 independent experiments)				
**Fig 4l area**	Unpaired *t*-test (Two-tailed)	t=5.645, df=22.02		WT vs. htr1b-/-	****	*P* < 0.0001
	Descriptive	WT: 57.63 ± 26.11 (n = 20 neurons examined over 20 independent experiments)				
		htr1b-/-: 23.33 ± 5.828 (n = 12 neurons examined over 12 independent experiments)				
**Suppl 1c**	One-way ANOVA	F(3, 24)=0.8758, P = 0.4674	Tukey's test	1 months vs. 2 months	ns	*Padj* = 0.8737
				1 months vs. 3 months	ns	*Padj* = 0.8684
				1 months vs. 4 months	ns	*Padj* = 0.9998
				2 months vs. 3 months	ns	*Padj* = 0.3907
				2 months vs. 4 months	ns	*Padj* = 0.9077
				3 months vs. 4 months	ns	*Padj* = 0.833
	Descriptive	1 months: 21.17 ± 2.563 (n = 6 fish)				
		2 months: 19.70 ± 2.791 (n = 10 fish)				
		3 months: 22.83 ± 5.529 (n = 6 fish)				
		4 months: 21.00 ± 1.099 (n = 6 fish)				
**Suppl 1h**	Unpaired *t*-test (Two-tailed)	t=0.3813, df=8		Ctrl: Seg5. vs. Seg.12.	ns	*P* = 0.7129
	Unpaired *t*-test with Welch's correction (Two-tailed)	t=5.570, df=5.109		1wpi: Seg5. vs. Seg.12.	**	*P* = 0.0024
	Descriptive	Ctrl Seg5.: 21.96 ± 2.284 (n = 5 fish)				
		Ctrl Seg12.: 21.40± 2.360 (n = 5 fish)				
		1wpiSeg5.: 26.62 ± 10.76 (n = 6 fish)				
		1wpi Seg12.: 2.007± 1.125 (n = 6 fish)				
**Suppl 1i**	Descriptive	1-cut injury seg.: 41.50± 8.699 (n = 4 fish)				
		2-cut injury seg.: 15.56 ± 6.167 (n = 9 fish)				
		1-cut distal seg.: 1.500 ± 1.732 (n = 4 fish)				
		2-cut distal seg.: 0.5556 ± 0.5270 (n = 9 fish)				
		uninjured: 0 ± 0 (n = 6 fish)				
**Suppl 1k mCherry+ cell**	Unpaired *t*-test (Two-tailed)	t=0.1523, df=20		MTZ- vs. MTZ+	ns	*P* = 0.8805
	Descriptive	MTZ-: 63.21 ± 25.40 (n = 14 fish)				
		MTZ+: 61.50 ± 25.40 (n = 8 fish)				
**Suppl 1k Free swimming**	Unpaired *t*-test (Two-tailed)	t=0.5941, df=31		MTZ- vs. MTZ+	ns	*P* = 0.5568
	Descriptive	MTZ-: 2.134 ± 0.9774 (n = 20 fish)				
		MTZ+: 1.937 ± 0.8545 (n = 13 fish)				
**Suppl 1k Max speed**	Unpaired *t*-test (Two-tailed)	t=0.1341, df=37		MTZ- vs. MTZ+	ns	*P* = 0.8940
	Descriptive	MTZ-: 9.167 ± 3.726 (n = 24 fish)				
		MTZ+: 9.000 ± 3.854 (n = 15 fish)				
**Suppl 1k D488+ cell**	Unpaired *t*-test (Two-tailed)	t=0.007711, df=20		MTZ- vs. MTZ+	ns	*P* = 0.9939
	Descriptive	MTZ-: 32.29 ± 8.543 (n = 14 fish)				

		MTZ+: 32.25 ± 13.29 (n = 8 fish)				
**Suppl 1k D488+ axon**	Unpaired *t*-test (Two-tailed)	t=1.143, df=17		MTZ- vs. MTZ+	ns	*P* = 0.2690
	Descriptive	MTZ-: 17.85 ± 6.818 (n = 11 fish)				
		MTZ+: 20.92 ± 3.844 (n = 8 fish)				
**Suppl 2c**	Descriptive	Resting: -62.20 ± 3.485 (n = 7 neurons)				
		Firing threshold: -32.56 ± 8.621 (n = 7 neurons)				
		Input resistance: 1.489 ± 0.4059 (n = 7 neurons)				
**Suppl 2g soma amplitude**	One-way ANOVA	F(2, 95.68)=28.64, P < 0.0001	Dunnett's T3 test	uninjured Ctrl vs. distal seg.	ns	*Padj* = 0.9271
				uninjured Ctrl vs. injuryl seg.	****	*Padj* < 0.0001
				distal seg. Ctrl vs. injuryl seg.	****	*Padj* < 0.0001
	Descriptive	uninjured Ctrl: 12.55 ± 7.343 (n = 30 neurons examined over 3 independent experiments)				
		distal seg.: 11.44 ± 8.448 (n = 31 neurons examined over 8 independent experiments)				
		injuryl seg.: 25.85 ± 11.18 (n = 40 neurons examined over 8 independent experiments)				
**Suppl 2g soma frequency**	One-way ANOVA	F(2, 98)=11.99, P < 0.0001	Tukey's test	uninjured Ctrl vs. distal seg.	ns	*Padj* = 0.7048
				uninjured Ctrl vs. injuryl seg.	****	*Padj* < 0.0001
				distal seg. Ctrl vs. injuryl seg.	**	*Padj* = 0.0011
	Descriptive	uninjured Ctrl: 0.02019 ± 0.01096 (n = 30 neurons examined over 3 independent experiments)				
		distal seg.: 0.02258 ± 0.01337 (n = 31 neurons examined over 8 independent experiments)				
		injuryl seg.: 0.03292 ± 0.01085 (n = 40 neurons examined over 8 independent experiments)				
**Suppl 2g terminal amplitude**	One-way ANOVA	F(2, 63.83)=99.08, P < 0.0001	Dunnett's T3 test	uninjured Ctrl vs. distal seg.	ns	*Padj* = 0.9997
				uninjured Ctrl vs. injuryl seg.	****	*Padj* < 0.0001
				distal seg. Ctrl vs. injuryl seg.	****	*Padj* < 0.0001
	Descriptive	uninjured Ctrl: 10.38 ± 4.382 (n = 30 neurons examined over 3 independent experiments)				
		distal seg.: 10.28 ± 5.289 (n = 31 neurons examined over 8 independent experiments)				
		injuryl seg.: 33.80 ± 12.96 (n = 40 neurons examined over 8 independent experiments)				
**Suppl 2g terminal frequency**	One-way ANOVA	F(2, 98)=45.02, P < 0.0001	Tukey's test	uninjured Ctrl vs. distal seg.	ns	*Padj* = 0.9421
				uninjured Ctrl vs. injuryl seg.	****	*Padj* < 0.0001
				distal seg. Ctrl vs. injuryl seg.	****	*Padj* < 0.0001
	Descriptive	uninjured Ctrl: 10.38 ± 4.382 (n = 30 neurons examined over 3 independent experiments)				
		distal seg.: 10.28 ± 5.289 (n = 31 neurons examined over 8 independent experiments)				
		injuryl seg.: 33.80 ± 12.96 (n = 40 neurons examined over 8 independent experiments)				
**Suppl 2j**	Descriptive	Glutmate: 6.250 ± 3.804 (n = 10 fish)				
		GABA: 6.250 ± 4.710 (n = 10 fish)				
		ChAT: 0.6098 ± 1.220 (n = 4 fish)				
**Suppl 3b**	Unpaired *t*-test (Two-tailed)	t=12.75, df=4		WT vs. htr1b-/-	***	*P* = 0.0002
	Descriptive	WT: 1.004 ± 0.1106 (n = 3 technical replicates of 20 pooled fish, the experiment was performed in triplicate)				
		htr1b-/-: 0.1606 ± 0.02988 (n = 3 technical replicates of 20 pooled fish, the experiment was performed in triplicate)				
**Suppl 3c**	Unpaired *t*-test (Two-tailed)	t=7.023, df=4		WT vs. htr2b-/-	**	*P* = 0.0022
	Descriptive	WT: 1.002 ± 0.06810 (n = 3 technical replicates of 20 pooled fish, the experiment was performed in triplicate)				
		htr2b-/-: 0.6884 ± 0.03643 (n = 3 technical replicates of 20 pooled fish, the experiment was performed in triplicate)				

**Suppl 3d**	Unpaired *t*-test with Welch's correction (Two-tailed)	t=70.36, df=2.018	WT vs. htr7c-/-	***	*P* = 0.0002
	Descriptive	WT: 1.000 ± 0.01745 (n = 3 technical replicates of 20 pooled fish, the experiment was performed in triplicate)			
		htr7c-/-: 0.2898 ± 0.001160 (n = 3 technical replicates of 20 pooled fish, the experiment was performed in triplicate)			
**Suppl 3e**	Unpaired *t*-test (Two-tailed)	t=0.1186, df=12	WT vs. htr1b-/-	ns	*P* = 0.9075
	Descriptive	WT: 57.50 ± 25.38 (n = 8 fish)			
		htr1b-/-: 58.83 ± 11.75 (n = 6 fish)			
**Suppl 3f 5HT+ cell**	Unpaired *t*-test (Two-tailed)	t=1.914, df=17	WT vs. *htr2b-/-*	ns	*P* = 0.0726
	Unpaired *t*-test (Two-tailed)	t=0.5135, df=19	WT vs. *htr7c-/-*	ns	*P* = 0.6135
	Descriptive	WT: 38.00 ± 12.27 (n = 9 fish)			
		htr2b-/-: 29.50 ± 6.536 (n = 10 fish)			
		htr7c-/-: 35.42 ± 10.74 (n = 12 fish)			
**Suppl 3f Free swimming**	Unpaired *t*-test (Two-tailed)	t=2.387, df=19	WT vs. *htr2b-/-*	*	*P* = 0.0275
	Unpaired *t*-test with Welch's correction (Two-tailed)	t=1.871, df=11.12	WT vs. *htr7c-/-*	ns	*P* = 0.0879
	Descriptive	WT: 3.638 ± 1.968 (n = 10 fish)			
		htr2b-/-: 2.000 ± 1.095 (n = 11 fish)			
		htr7c-/-: 2.407 ± 0.7402 (n = 12 fish)			
**Suppl 3f Max speed**	Unpaired *t*-test (Two-tailed)	t=1.181, df=23	WT vs. *htr2b-/-*	ns	*P* = 0.2497
	Unpaired *t*-test (Two-tailed)	t=0.3763, df=22	WT vs. *htr7c-/-*	ns	*P* = 0.7103
	Descriptive	WT: 11.00 ± 2.954 (n = 12 fish)			
		htr2b-/-: 9.308 ± 4.070 (n = 13 fish)			
		htr7c-/-: 11.50 ± 3.529 (n = 12 fish)			
**Suppl 3f RD+ cell**	Unpaired *t*-test (Two-tailed)	t=2.091, df=19	WT vs. *htr2b-/-*	ns	*P* = 0.0502
	Unpaired *t*-test with Welch's correction (Two-tailed)	t=3.125, df=9.735	WT vs. *htr7c-/-*	*	*P* = 0.0111
	Descriptive	WT: 39.20 ± 14.91 (n = 10 fish)			
		htr2b-/-: 26.27 ± 13.42 (n = 11 fish)			
		htr7c-/-: 24.17 ± 3.298 (n = 12 fish)			
**Suppl 3f RD+ axon**	Unpaired *t*-test (Two-tailed)	t=0.9887, df=24	WT vs. *htr2b-/-*	ns	*P* = 0.3327
	Unpaired *t*-test (Two-tailed)	t=1.035, df=25	WT vs. *htr7c-/-*	ns	*P* = 0.0111
	Descriptive	WT: 21.47 ± 11.11 (n = 15 fish)			
		htr2b-/-: 17.56 ± 8.091 (n = 11 fish)			
		htr7c-/-: 25.55 ± 8.843 (n = 12 fish)			

**Suppl 3g 5HT+ cell**	Unpaired *t*-test (Two-tailed)	t=0.4860, df=14	DMSO vs. CP93129	ns	*P* = 0.6345
	Descriptive	DMSO: 60.71 ± 16.63 (n = 7 fish)			
		CP93129: 66.11 ± 25.34 (n = 9 fish)			
**Suppl 3g free swimming**	Unpaired *t*-test (Two-tailed)	t=2.290, df=14	DMSO vs. CP93129	*	*P* = 0.0381
	Descriptive	DMSO: 2.164 ± 0.6382 (n = 7 fish)			
		CP93129: 2.908 ± 0.6499 (n = 9 fish)			
**Suppl 3g max speed**	Unpaired *t*-test (Two-tailed)	t=2.209, df=16	DMSO vs. CP93129	*	*P* = 0.0421
	Descriptive	DMSO: 11.00 ± 3.854 (n = 8 fish)			
		CP93129: 14.40 ± 2.675 (n = 10 fish)			
**Suppl 3g RD+ cell**	Unpaired *t*-test (Two-tailed)	t=2.442, df=14	DMSO vs. CP93129	*	*P* = 0.0285
	Descriptive	DMSO: 39.05 ± 19.36 (n = 7 fish)			
		CP93129: 57.12 ± 9.807 (n = 9 fish)			
**Suppl 3g RD+ axon**	Unpaired *t*-test (Two-tailed)	t=2.606, df=14	DMSO vs. CP93129	*	*P* = 0.0207
	Descriptive	DMSO: 17.35 ± 6.636 (n = 7 fish)			
		CP93129: 27.54 ± 8.510 (n = 9 fish)			
