# Supplementary material for: An injury-induced serotonergic neuron subpopulation contributes to axon regrowth and function restoration after spinal cord injury in zebrafish
Source: Nat Commun. 2021 Dec 7;12:7093. doi: 10.1038/s41467-021-27419-w (PMC8651775; doi:10.1038/s41467-021-27419-w)
Supplement: Supplementary file 3 — Description of Additional Supplementary Files [file 41467_2021_27419_MOESM3_ESM.docx]

Description of Additional Supplementary Files

Title: Supplementary Video 1

Description: Free swimming recordings of wild-type uninjured, wild-type 2 wpi and htr1b-/- 2 wpi zebrafish. The drawings at the bottom-left of each dish in the video indicate the head angle and tail angle. Straight red lines represent the body positions without any movement. Green dots represent the body binding points when the maximum body curvatures were observed. α represent head binding angle. β represent tail binding angle. For the uninjured animal, head binding angle is smaller than the tail angle; for the wild-type 2 wpi animal, head binding angle is similar to the tail angle; for the htr1b-/- 2 wpi animal, head binding angle is larger than the tail angle.
