# Supplementary material for: An injury-induced serotonergic neuron subpopulation contributes to axon regrowth and function restoration after spinal cord injury in zebrafish
Source: Nat Commun. 2021 Dec 7;12:7093. doi: 10.1038/s41467-021-27419-w (PMC8651775; doi:10.1038/s41467-021-27419-w)
Supplement: Supplementary file 5 — Reporting Summary [file 41467_2021_27419_MOESM5_ESM.pdf]

nature research

Corresponding author(s): Jianren Song

Last updated by author(s): Nov 8, 2021

Reporting Summary

Nature Research wishes to improve the reproducibility of the work that we publish. This form provides structure for consistency and transparency in reporting. For further information on Nature Research policies, see our Editorial Policies and the Editorial Policy Checklist.

Statistics

For all statistical analyses, confirm that the following items are present in the figure legend, table legend, main text, or Methods section.

n/a	Confirmed	
☐	☒	The exact sample size (*n*) for each experimental group/condition, given as a discrete number and unit of measurement
☐	☒	A statement on whether measurements were taken from distinct samples or whether the same sample was measured repeatedly
☐	☒	The statistical test(s) used AND whether they are one- or two-sided<br>*Only common tests should be described solely by name; describe more complex techniques in the Methods section.*
☒	☐	A description of all covariates tested
☐	☒	A description of any assumptions or corrections, such as tests of normality and adjustment for multiple comparisons
☐	☒	A full description of the statistical parameters including central tendency (e.g. means) or other basic estimates (e.g. regression coefficient) AND variation (e.g. standard deviation) or associated estimates of uncertainty (e.g. confidence intervals)
☒	☐	For null hypothesis testing, the test statistic (e.g. *F*, *t*, *r*) with confidence intervals, effect sizes, degrees of freedom and *P* value noted<br>*Give P values as exact values whenever suitable.*
☒	☐	For Bayesian analysis, information on the choice of priors and Markov chain Monte Carlo settings
☒	☐	For hierarchical and complex designs, identification of the appropriate level for tests and full reporting of outcomes
☒	☐	Estimates of effect sizes (e.g. Cohen's *d*, Pearson's *r*), indicating how they were calculated

Our web collection on statistics for biologists contains articles on many of the points above.

Software and code

Policy information about availability of computer code

Data collection	pClamp v10, SciScan 1.2 software, CoreView software v2.1, Summit v6.3
Data analysis	Prism v8.0, Fiji v1.53c, Matlab R2018a v9.4, Zebrazoom v1.17, Clampfit v10.6

For manuscripts utilizing custom algorithms or software that are central to the research but not yet described in published literature, software must be made available to editors and reviewers. We strongly encourage code deposition in a community repository (e.g. GitHub). See the Nature Research guidelines for submitting code & software for further information.

Data

Policy information about availability of data

All manuscripts must include a data availability statement. This statement should provide the following information, where applicable:

- Accession codes, unique identifiers, or web links for publicly available datasets
- A list of figures that have associated raw data
- A description of any restrictions on data availability

All relevant data supporting this study are available from the corresponding authors upon reasonable request. Raw and processed RNA-seq data generated in this study are deposited into the GEO database with accession number GSE182911 [https://www.ncbi.nlm.nih.gov/geo/query/acc.cgi?acc=GSE182911]. Source data are provided with this paper.

Field-specific reporting

Please select the one below that is the best fit for your research. If you are not sure, read the appropriate sections before making your selection.

- [x] Life sciences
- [ ] Behavioural & social sciences
- [ ] Ecological, evolutionary & environmental sciences

For a reference copy of the document with all sections, see nature.com/documents/nr-reporting-summary-flat.pdf

Life sciences study design

All studies must disclose on these points even when the disclosure is negative.

Sample size	Sample-size is estimated base on 3R principles, how large a difference, how much variability,"p" value and the confidence intervals. We used online spreadsheet (Lamorte's Power Calculations) to estimate the sample size.
Data exclusions	No data were excluded from the analyses.
Replication	At least three measures were taken to verify the reproducibility of the experimental findings. All attempts at replication were successful.
Randomization	All samples were randomly allocated into experimental groups.
Blinding	The investigators were blinded to group allocation during data collection and analysis.

Reporting for specific materials, systems and methods

We require information from authors about some types of materials, experimental systems and methods used in many studies. Here, indicate whether each material, system or method listed is relevant to your study. If you are not sure if a list item applies to your research, read the appropriate section before selecting a response.

Materials & experimental systems

n/a	Involved in the study	
[ ]	[x]	Antibodies
[x]	[ ]	Eukaryotic cell lines
[x]	[ ]	Palaeontology and archaeology
[ ]	[x]	Animals and other organisms
[x]	[ ]	Human research participants
[x]	[ ]	Clinical data
[x]	[ ]	Dual use research of concern

Methods

n/a	Involved in the study	
[x]	[ ]	ChIP-seq
[ ]	[x]	Flow cytometry
[x]	[ ]	MRI-based neuroimaging

Antibodies

Antibodies used

1. Anti-GFP antibody [9F9.F9], Supplier Name: Abcam, Cat#: ab1218, Clone ID: 9F9.F9, lot#: GR213436-49, 1:2000
2. Anti-GFP antibody, Supplier Name: Abcam, Cat#: ab290, lot#: GR3251545-1, 1:1000
3. Rabbit Anti-Serotonin Antibody, Unconjugated, Supplier Name: Sigma-Aldrich, Cat#: S5545, lot#: 087M4758V 1:3000
4. Anti-Digoxigenin-AP Fab fragments antibody Sigma-Aldrich Cat# 11093274910, lot#: 32871920, 1:3000
5. NeuN antibody, Supplier Name: Proteintech, Cat#: 26975-1-AP, lot#: 00050791, 1:200
6. Anti-Choline Acetyltransferase antibody, Supplier Name: Millipore, Cat# AB144P, lot#: 3475626, 1:100
7. Anti-GABA antibody produced in rabbit, Supplier Name: Sigma-Aldrich, Cat# A2052, lot#: 047M4852V, 1:1000
8. Anti-Glutamate antibody produced in rabbit, Supplier Name: Sigma-Aldrich, Cat# G6642, lot#: 108M4860V, 1:2000

9 Donkey Anti-Mouse IgG (H+L) Polyclonal Antibody, Alexa Fluor 647 Conjugated, Supplier Name: Invitrogen, Cat#: A-31571, lot#: 1900251, 1:500

10. Alexa Fluor 488-AffiniPure Donkey Anti-Mouse IgG (H+L) antibody, Supplier Name: Jackson ImmunoResearch Labs, Cat#: 715-545-150, lot#: 138609, 1:200
11. Alexa Fluor 488-AffiniPure Donkey Anti-Rabbit IgG (H+L) antibody, Supplier Name: Jackson ImmunoResearch Labs, Cat#: 711-545-152, lot#: 144917, 1:200
12. DyLight 405-AffiniPure Goat Anti-Rabbit IgG (H+L) antibody, Supplier Name: Jackson ImmunoResearch Labs, Cat#: 111-475-003, lot#: 135289, 1:200

Validation

1. Anti-GFP antibody [9F9.F9] (Cat#:ab1218); species: species independent; application: WB, IHC-Fr, Sandwich ELISA, ICC/IF; manufacturer's website: https://www.abcam.cn/gfp-antibody-9f9f9-ab1218.html

2.Anti-GFP antibody (Cat#:ab290); species: species independent; application: ELISA, IHC-FrFl, Electron Microscopy, HHC-FoFr, ICC, IHC-P, IHC-Fr, IP, WB; manufacturer's website: https://www.abcam.cn/gfp-antibody-ab290.html

3. Rabbit Anti-Serotonin Antibody (Cat#: S5545); species: human, rat; application: immunohistochemistry (formalin-fixed, paraffin-

embedded sections), immunohistochemistry (frozen sections); manufacturer's website: https://www.sigmaaldrich.com/catalog/product/sigma/s5545?lang=zh&region=CN
4. Anti-Digoxigenin-AP Fab fragments antibody (Cat# 11093274910); application: cDNA array, Colony/plaque hybridization, Dot blot, ELISA, Gel shift assay, Immunohistocytochemistry, In situ hybridization, Nonradioactive DNA sequencing blot, Northern blot, RNase protection assay, Southern blot, Western blot, Fluorescent in situ hybridization, Section in situ hybridization and whole mount in situ hybridization, Electrophoretic mobility shift assay; manufacturer's website: https://www.sigmaaldrich.cn/CN/en/product/roche/11093274910?context=product
5. NeuN antibody (Cat#: 26975-1-AP); species: Human, Mouse, Rat, Pig; application: WB, IHC, IF; manufacturer's website: https://www.ptglab.com/products/NeuN-Antibody-26975-1-AP.htm
6. Anti-Choline Acetyltransferase antibody (Cat# AB144P); species: guinea pig, mouse, opossum, zebrafish (THE JOURNAL OF COMPARATIVE NEUROLOGY 474:75–107 (2004).), avian, rat, chicken, monkey, human; application: immunocytochemistry, western blot; manufacturer's website: https://www.sigmaaldrich.cn/CN/zh/product/mm/ab144p?context=product
7. Anti-GABA antibody produced in rabbit (Cat# A2052); species: wide range, rat, Drosophila; application: immunocytochemistry, dot blot; manufacturer's website: https://www.sigmaaldrich.cn/CN/zh/product/sigma/a2052?context=product
8. Anti-Glutamate antibody produced in rabbit (Cat# G6642); species: wide range; application: dot blot: 1:15,000; manufacturer's website: https://www.sigmaaldrich.cn/CN/zh/product/sigma/g6642?context=product

Animals and other organisms

Policy information about studies involving animals; ARRIVE guidelines recommended for reporting animal research

Laboratory animals

Young adult zebrafish of both gender between six to eight weeks old of 1.5-2.0 cm in length were used.
1. Wild-type zebrafish AB line;
2. Tg(tph2:GFP)
3. Tg(tph2:Gal4)
4. Tg(UAS:GCaMP6)
5. Tg(UAS:nfsB-mcherry)
6. Tg(vglut2a:Gal4;UAS:GFP)
7. Tg(glyt2:GFP)
8. Tg(UAS:mcherry)

Wild animals

The study did not involve wild animals.

Field-collected samples

The study did not involve samples collected from the field.

Ethics oversight

All the experimental protocols were approved by the Animal Use Committee of Tongji University.

Note that full information on the approval of the study protocol must also be provided in the manuscript.

Flow Cytometry

Plots

Confirm that:

☐ The axis labels state the marker and fluorochrome used (e.g. CD4-FITC).

☐ The axis scales are clearly visible. Include numbers along axes only for bottom left plot of group (a 'group' is an analysis of identical markers).

☐ All plots are contour plots with outliers or pseudocolor plots.

☐ A numerical value for number of cells or percentage (with statistics) is provided.

Methodology

Sample preparation

Spinal cord tissue were dissected out from the fish, and digested using the Papain kit (Worthington Biochemical, 10U/ml in DMEM, osmolarity of 285 mOsm) on a mechanical shaker at 37°C, 300 rpm for 15 min and terminated by adding DMEM. Then the tissues were further dissociated by gentle pipetting up and down for 5 min. The resulting cell solution was filtered with a 40 µm cell strainer (BD Falcon) and Draq5 (Thermo Scientific) was added to check the viability. Subsequently, fluorescence activated cell sorting (FACS) was performed using the Beckman Coulter Moflo Astrios Cell Sorter (Beckman Coulter). The isolated cells were collected into PBS.

Instrument

Beckman Coulter Moflo Astrios Cell Sorter (Beckman Coulter)

Software

Summit V6.3.1

Cell population abundance

Transgenic lines with target cells labeled by GFP were used for fluorescence activated cell sorting. Draq5 (Thermo Scientific) was added to check the viability. Live cells showing strong fluorescence signal was sorted out, which account for about 1% within the whole population including fractions. To guarantee the purity of the samples, the instrument was adjusted to gain high sensitivity with CV value being less than 3. Meanwhile, the purity model was chosen for sorting with the grading being 1.0.

Gating strategy	We first set the gates for starting cell population according to the FSC and SSC characteristics which was assigned as region 1. After that, we eliminate cell adhesion according to the FSC which was assigned as region2. Then the gate for live cells were set according to the detection of Draq5 labeling, which was assigned as region 3. At last, GFP+/DR- and GFP+/DR+ cell populations were isolated according to the fluorescence signal.

☐ Tick this box to confirm that a figure exemplifying the gating strategy is provided in the Supplementary Information.
